# Physical activity affects dysthyreosis by thyroid hormones sensitivity: a population-based study

**DOI:** 10.3389/fendo.2024.1418766

**Published:** 2024-10-28

**Authors:** Shu-yang Zhang, Xue-qing Hu, Cheng Xiang, Tao Xiang, Song-xue Guo, Fei-hu Zhi, Ping Zhao, Jia-yan Zhu, Chen-yang Zhang

**Affiliations:** ^1^ Department of General Surgery, Shaoxing Hospital of Traditional Chinese Medicine [Shaoxing Traditional Chinese Medicine (TCM) Hospital Affiliated to Zhejiang Chinese Medical University], Shaoxing, Zhejiang, China; ^2^ Department of Plastic Surgery, The Second Affiliated Hospital of Zhejiang University School of Medicine, Hangzhou, Zhejiang, China; ^3^ Department of Thyroid Surgery, The Second Affiliated Hospital of Zhejiang University School of Medicine, Hangzhou, Zhejiang, China; ^4^ Department of Colorectal Surgery, The First Affiliated Hospital of Zhejiang University School of Medicine, Hangzhou, Zhejiang, China; ^5^ XiangYa School of Medicine, Central South University, Changsha, Hunan, China

**Keywords:** physical activity, thyroid feedback quantile-based index, sensitivity, dysthyreosis, metabolic equivalent task, hypothalamic pituitary thyroid axis

## Abstract

**Objective:**

Physical activity (PA) plays an important role in human health. However, the relationship between the PA and dysthyreosis was not clear. This study aimed to explore this question.

**Methods:**

We performed a population-based study on the basis of the participant’s information that was collected from the National Health and Nutrition Examination Survey (NHANES) database. The association of the thyroid hormone and total PA metabolic equivalent task (MET) were assessed via linear regression analysis with adjusting for different covariates. Moreover, we also used path analysis to explore the causality between PA, thyroid hormone index, and dysthyreosis. The restricted cubic spline analysis was used to explore the non-linear relationship between the thyroid hormone index and the PA total MET.

**Results:**

A total of 2118 participants aged≥20, including 969 females and 1149 males, were collected from NHANES. The linear regression with multivariate adjustment suggested a linear relationship between the PA total MET and Thyroid-Stimulating Hormone Index (TSHI), and Thyroid Feedback Quantile-Based Index (TFQI), respectively. And TFQI and dysthyresis occurrence were significantly correlated (P<0.05). The path analysis showed that the PA total MET affected dysthyreosis occurrence by TFQI. In addition, we found a non-linear relationship between the total PA met and dysthyreosis via restricted cubic spline analysis.

**Conclusions:**

PA was significantly correlated with dysthyreosis via thyroid hormone sensitivity. Therefore, it can be considered to prevent the occurrence of dysthyreosis by regulating thyroid hormone sensitivity through PA in daily life.

## 1 Introduction

Physical activity (PA), any bodily movement with energy expenditure produced via skeletal muscles, crucially affects human health and well-being in daily life (1). PA is related to many human diseases such as obesity, type 2 diabetes, depression, and cardiovascular diseases (CVD). Most studies indicated that regular PA reduced the risk of those chronic diseases (2–4). Higher PA was strongly associated with CVD patients’ mortality (5). Matthew et al. suggested that significant mental health can benefit from being physically active via meta-analysis (6). Most diseases were related to abnormalities in the physiological processes of energy metabolism regulated by thyroid hormones (7–10).

Thyroid hormones play a key role in the human endocrine system, affecting energy expenditure via regulating cellular respiration and thermogenesis and further affecting the resting metabolic rate (11). Free triiodothyronine (FT3), Free thyroxine (FT4), and Thyroid-stimulating Hormone (TSH) are classical indicators for evaluating thyroid function. Thyroid-stimulating Hormone Index (TSHI), Thyrotroph Thyroxine Resistance Index (TT4RI), and Thyroid Feedback Quantile-based index (TFQI) provide a new insight for assessing the degree of negative feedback between the FT3, FT4 and TSH (12–14). There were some studies about the influence of PA on thyroid hormone levels. Ronny et al. found that sub-lactate threshold training accelerated the TSH-mediated signaling pathway in the skeletal muscle of male rats (5). Krogh et al. found that four months of vigorous PA decreased the levels of FT3, FT4, and TSH in the dogs (15). Christopher et al. indicated that daily PA was negatively associated with thyroid hormone levels among adults (16). It followed that PA was an important factor influencing the Thyroid-related hormone secretion.

Thyroid-related hormone levels and thyroid hormone sensitivity are indicators for evaluating thyroid function. However, the population-based research about the relationship between PA and thyroid hormone sensitivity is limited, and the influence of PA on the dysthyreosis of humans remains unclear. Hence, in the present study, we hypothesized that PA was independently associated with the occurrence of dysthyreosis. We predicted that PA affected thyroid function by affecting thyroid hormone levels or sensitivity. Therefore, we collected the related data from the National Health and Nutrition Examination Survey (NHANES) database to explore the association of PA total MET with dysthyreosis and fill this gap in this field.

## 2 Materials and methods

### 2.1 Participants

The information of participants was collected from 2007-2012 continuous biennial cycles of the U.S. NHANES which is a nationwide, ongoing, cross-sectional, multistage survey conducted by the U.S. Centers for Disease Control and Prevention (CDC) [CDC, National Health and Nutrition Examination Survey. National Center for Health Statistics, Centers for Disease Control and Prevention (2022)]. The project is to accurately assess the health and nutritional status of Americans.

**Figure 1:**
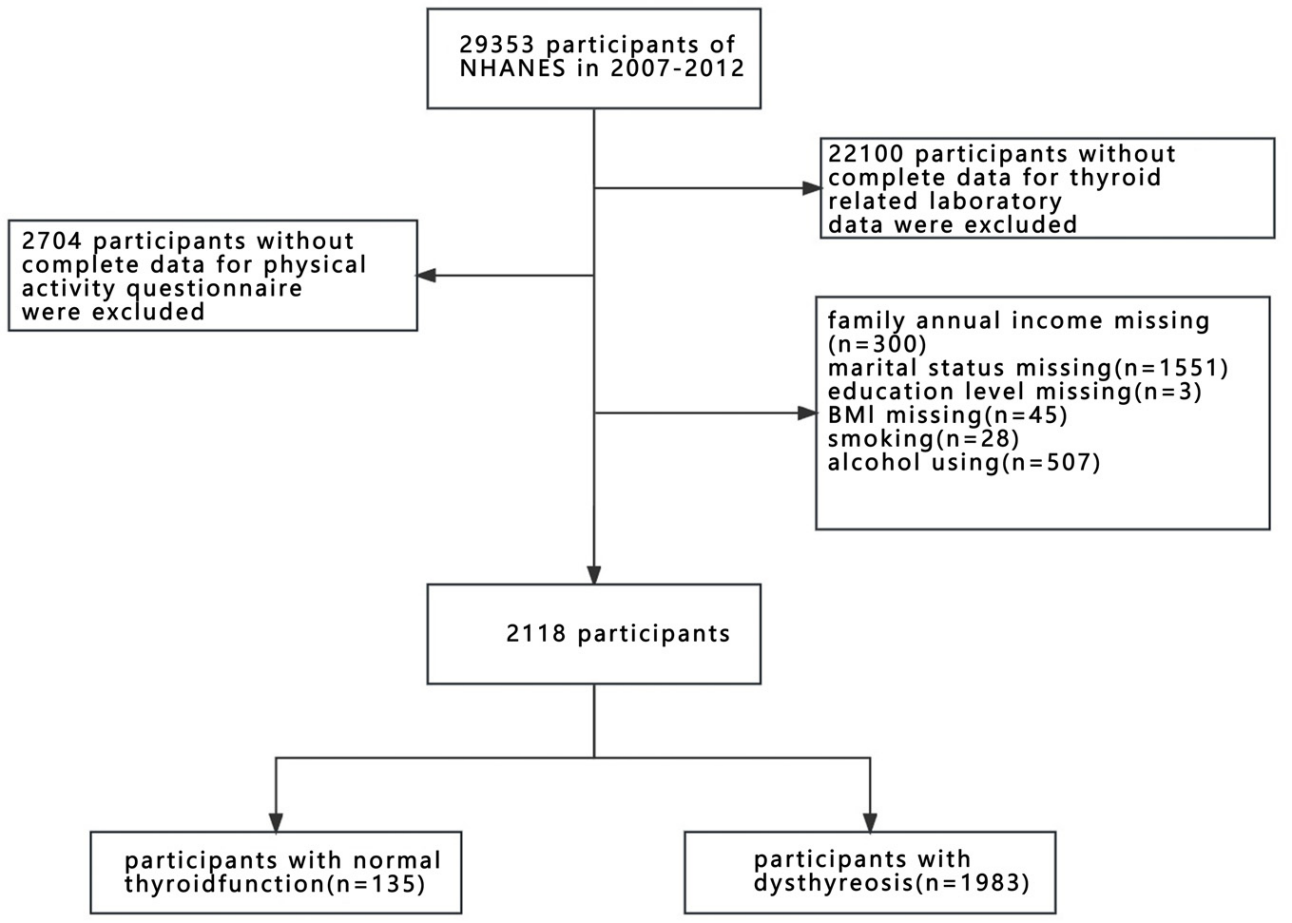
Researches flow chart for inclusion and exclusion of participants.

A total of 29353 participants from 2007-2012 were obtained for further screening. The excluded criteria included (1) the participants without complete PA questionnaire; (2) the participants without complete thyroid-related laboratory data, and (3) the participants missing covariates data (demographic characteristic: age, sex, race, marital status, family annual income, education level, body mass index (BMI); lifestyle: smoking, alcohol using; health status: hypertension, diabetes, stroke). The inclusion criteria included (1) the participants with a complete PA questionnaire; (2) the participants with complete thyroid-related laboratory data, (3) the participants with complete covariates (demographic characteristics: age, sex, race, marital status, family annual income, BMI, education level; lifestyle: smoking, alcohol using; health status: hypertension, diabetes, stroke). Finally, 2118 participants were included in the study. It is worth noting that health status may affect the results of the study, so we included more important and common variables: hypertension, diabetes, and stroke. The specific screening process is shown in **Figure 1**.

### 2.2 Measurement of hormone content and sensitivity to thyroid hormone indices

FT3, Total T3 (TT3), Total T4 (TT4), and FT4, TSH were measured via the thyroid blood sample at the University of Washington in Seattle. The methods of measurement were a two-site immunoenzymatic (“sandwich”) assay and a competitive binding immunoenzymatic assay. The specific experimental steps were presented on the NHANES official website (Method for determination of thyroid hormone in NHANES 2007-2008, https://wwwn.cdc.gov/Nchs/Nhanes/2007-2008/THYROD_E.htm, accessed July 2023). TSHI, TT4RI, and TFQI were calculated using the following formula, respectively.


TSHI = InTSH (mUI/L) + 0.1345 × FT4 (pmol/L)

TT4RI = FT4 (pmol/L) × TSH (mUI/L)

TFQI = cdf FT4 + cdf TSH−1

CDF: Cumulative distribution function

### 2.3 Dysthyreosis diagnosis

The dysthyreosis included hyperthyroidism, subclinical hyperthyroidism, hyperthyroidism, and subclinical hypothyroidism. Hence, the four kinds of dysthyreoses were assessed via TSH content and FT4 content. TSH<0.45 mUI/L and FT4>1.6 ng/dL were considered hyperthyroidism. TSH>4.5 mUI/L and FT4<0.6 ng/dL were considered hypothyroidism. Sub-clinical hyperthyroidism was diagnosed according to TSH<0.45 mUI/L and 0.6<FT4<1.6 ng/dL, and subclinical hypothyroidism was diagnosed according to TSH>4.5 mUI/L and 0.6<FT4<1.6 ng/dL (17).

### 2.4 PA total metabolic equivalent task score computation

Each participant completed a PA questionnaire, which included questions related to all PA performed in the past 30 days. This questionnaire recorded the type, duration, intensity, and frequency of activities, including transportation, occupational, and leisure activities. Activity intensity was categorized into moderate and vigorous activities. Moderate-intensity activities were defined as those that induced light sweating or slight to moderate breathing or heart rate increases. Vigorous activities were defined as those causing heavy sweating or substantial breathing or heart rate increases. The MET score for specific activities was calculated based on the type and intensity of the activity (18). Then, the weekly total PA volume was the sum of the work activity MET score, recreational activity MET score, and walk/bicycle activity MET score in one week. Every kind of PA MET score was computed via the following formula: Total MET (minutes per week) = number of days *number of minutes* MET scores.

### 2.5 Other participants’ characteristics

In this study, we collected the patients’ demographic characteristics including age (years), sex (male, female), race (non-Hispanic White, non-Hispanic Black, Mexican American, other Hispanic, and other Race - Including Multi-Racial), education (less than 9th grade, 9-11th Grade, high school grad/GED or equivalent, some college or AA degree, and college graduate or above), annual family income (< USD$20,000, USD$20,000–$75,000, and >USD$75,000), marital status (never marital status, marital status, divorced, living with a partner, widowed, and separated), BMI (<25 kg/m^2^, 25 kg/m^2^-30 kg/m^2^, and ≥30 kg/m^2^), and lifestyle in smoking and alcohol use. Smoking and drinking were classified according to the method based on the previous studies (19, 20). In addition, some disease information including hypertension, stroke, and diabetes was also collected. Hypertension was defined blood pressure (BP) of ≥140/90 mmHg. Diabetes was diagnosed based on the study of Xiao et al. (21). Stroke was diagnosed via the question: the doctor ever told you had a stroke?

### 2.6 Statistical analyses

All statistical analyses were performed using the ‘R’ software (version 4.2.2), and used the appropriate sample weights, 1/3 of two-year a subsample weights (WTSA2YR*1/3), on the basis of the NHANES analysis guidelines. p-value <0.05 was defined as statistically significant. Participants’ characteristics were divided into three groups based on the tertiles of PA total MET score (Q1, Q2, and Q3). Baseline categorical characteristics were expressed as frequencies and percentages, while continuous characteristics were reported as mean ± standard deviation. Subsequently, the difference between groups was compared using ANOVA for continuous characteristics, whereas the chi-square test was used to compare the categorical characteristics. The association between the thyroid hormone indexes and the PA total MET score was analyzed by linear regression. The relationship between the thyroid hormone indexes and the dysthyreosis status was analyzed via logistic regression. Moreover, we performed the three kinds of adjustment to assess the effect of the other characteristics. The relationship between the thyroid hormone index and the PA total MET was analyzed using restricted cubic spline analyses.

## 3 Results

### 3.1 Clinical characteristics

**Table 1** displays the baseline characteristics of 2118 participants aged≥20, stratified by three quantiles of PA total MET (Q1, Q2, and Q3). Several covariates showed significant differences among three groups including TSHI, TTSI, and TFQI age, sex, race, marital status, education level, smoking, alcohol use, hypertension, diabetes, and BMI (all P<0.05).

**Table 1:** Baseline characteristics.

Variable	PA total (MET-min/week)			P
	Q1 (n=713) 554.777 (14.428)	Q2 (n=699) 2181.743 (36.787)	Q3 (n=706) 10860.767 (318.146)	
age (years)	50.229 (0.754)	44.832 (0.979)	41.834 (1.066)	< 0.001
FT3 (pg/mL)	3.126 (0.021)	3.173 (0.027)	3.261 (0.023)	< 0.001
TSH (mIU/L)	1.944 (0.089)	2.178 (0.310)	1.765 (0.091)	0.326
FT4 (pmol/L)	10.852 (0.110)	10.644 (0.140)	10.216 (0.104)	< 0.001
TSHI	1.918 (0.037)	1.837 (0.035)	1.723 (0.036)	0.002
TT4RI	21.073 (0.965)	20.832 (1.444)	17.316 (0.669)	0.019
TFQI	0.013 (0.015)	-0.025 (0.018)	-0.095 (0.017)	< 0.001
age				< 0.001
≤60	458 (0.291)	430 (0.273)	586 (0.372)	
>60	255 (0.469)	169 (0.311)	120 (0.221)	
sex (n, %)				< 0.001
female	419 (61.306)	331 (48.605)	219 (30.908)	
male	294 (38.694)	368 (51.395)	487 (69.092)	
race (n, %)				< 0.001
non-Hispanic White	320 (70.068)	356 (76.443)	303 (67.985)	
non-Hispanic Black	164 (11.428)	112 (7.019)	156 (11.146)	
Mexican American	93 (6.506)	79 (5.728)	112 (9.020)	
other Hispanic	56 (4.375)	68 (5.271)	82 (7.017)	
other Race - Including Multi-Racial	80 (7.623)	84 (5.539)	53 (4.833)	
marital status (n, %)				< 0.001
never marital statusried	113 (14.197)	155 (20.912)	175 (24.262)	
marital statusried	345 (54.410)	370 (56.386)	347 (51.671)	
divorced	85 (11.278)	67 (9.861)	50 (8.629)	
living with partner	58 (9.368)	53 (7.116)	81 (10.127)	
widowed	88 (8.715)	37 (3.368)	23 (2.226)	
separated	24 (2.033)	17 (2.357)	30 (3.085)	
family annual income (n, %)				0.062
< USD$20,000	238 (22.416)	192 (19.449)	233 (25.115)	
USD$20,000–$75,000	294 (40.657)	309 (43.443)	313 (44.765)	
>USD$75,000	181 (36.927)	198 (37.108)	160 (30.120)	
education level (n, %)				< 0.001
less than 9th grade	61 (3.986)	47 (2.778)	62 (4.983)	
9-11th Grade	113 (12.276)	77 (6.626)	96 (9.976)	
high school grad/GED or equivalent	146 (21.380)	119 (16.156)	181 (26.789)	
some college or AA degree	217 (29.779)	208 (31.097)	236 (35.799)	
college graduate or above	176 (32.580)	248 (43.343)	131 (22.454)	
smoke (n, %)				< 0.001
former	189 (28.928)	184 (25.668)	151 (20.296)	
never	394 (52.960)	410 (60.202)	376 (55.855)	
now	130 (18.112)	105 (14.129)	179 (23.849)	
alcohol using (n, %)				0.004
former	120 (12.630)	92 (11.004)	85 (13.752)	
heavy	140 (22.104)	134 (20.923)	224 (30.770)	
mild	229 (33.418)	255 (37.950)	213 (30.366)	
moderate	113 (21.179)	136 (21.638)	124 (18.818)	
never	111 (10.668)	82 (8.484)	60 (6.294)	
hypertension (n, %)				0.026
no	384 (60.772)	451 (67.801)	479 (70.750)	
yes	329 (39.228)	248 (32.199)	227 (29.250)	
diabetes (n, %)				< 0.001
yes	202 (17.887)	147 (8.536)	110 (9.108)	
no	511 (77.402)	562 (84.713)	596 (86.814)	
stroke (n, %)				0.709
no	692 (98.347)	684 (98.716)	690 (98.306)	
yes	21 (1.653)	15 (1.284)	16 (1.694)	
BMI (n, %)				0.027
<25	206 (31.225)	241 (36.837)	223 (32.233)	
≥30	276 (38.344)	223 (28.298)	242 (31.968)	
25-30	231 (30.431)	235 (34.865)	241 (35.798)	

FT3, Free triiodothyronine; TSH, Thyroid Stimulating Hormone; FT4, Free Thyroxine; TSHI, Thyroid Stimulating Hormone Index; TT4RI, Thyrotroph Thyroxine Resistance Index; TFQI, Thyroid Feedback Quantile-based Index.

### 3.2 Association between thyroid hormone indexes and PA total MET score

To further explore the relationship between the PA total MET and thyroid hormone indexes, linear regression analyses were performed. The results showed that PA total MET was significantly related to the levels of TSHI, FT4, and TFQI, respectively both in crude and adjusted models (P<0.05, P for trend<0.05, **Table 2**).

**Table 2:** The association between PA total MET and 6 thyroid hormone indexes in the whole population by linear regression analyses (n=2118).

dependent variable	independent variable	crude model		adjusted model 1		adjusted model 2		adjusted model 3	
TT4RI	PAMET	95%CI	P	95%CI	P	95%CI	P	95%CI	P
	Q1	ref		ref		ref		ref	
	Q2	-0.241(-2.846, 2.363)	0.851	0.147(-2.370, 2.663)	0.905	0.117(-2.493, 2.727)	0.925	0.000(-3.269, 3.268)	1.000
	Q3	-3.757(-6.322, -1.191)	0.005	-2.736(-5.170, -0.302)	0.029	-2.551(-5.101, -0.002)	0.05	-2.598(-5.800, 0.604)	0.091
	P for trend		0.005		0.027		0.046		0.08
TSHI	Q1	ref		ref		ref		ref	
	Q2	-0.081(-0.158, -0.003)	0.041	-0.078(-0.153, -0.003)	0.042	-0.067(-0.146, 0.011)	0.088	-0.075(-0.166, 0.016)	0.087
	Q3	-0.195(-0.300, -0.089)	<0.001	-0.177(-0.292, -0.061)	0.004	-0.167(-0.287, -0.046)	0.010	-0.169(-0.320, -0.019)	0.034
	P for trend		<0.001		0.004		0.009		0.027
TFQI	Q1	ref		ref		ref		ref	
	Q2	-0.039(-0.084, 0.006)	0.09	-0.033(-0.075, 0.009)	0.121	-0.033(-0.076, 0.009)	0.115	-0.034(-0.084, 0.016)	0.145
	Q3	-0.108(-0.152, -0.064)	<0.001	-0.096(-0.142, -0.051)	<0.001	-0.093(-0.140, -0.046)	<0.001	-0.088(-0.147, -0.029)	0.012
	P for trend		<0.001		<0.001		<0.001		0.009
TSH	Q1	ref		ref		ref		ref	
	Q2	0.234(-0.336,0.803)	0.409	0.291(-0.350, 0.932)	0.358	0.271(-0.351, 0.893)	0.367	0.261(-0.497, 1.019)	0.416
	Q3	-0.179(-0.456,0.099)	0.199	-0.025(-0.349, 0.300)	0.876	0.011(-0.349, 0.371)	0.947	-0.011(-0.420, 0.398)	0.948
	P for trend		0.193		0.848		0.959		0.932
FT3	Q1	ref		ref		ref		ref	
	Q2	0.047(-0.008,0.102)	0.089	-0.021(-0.074, 0.032)	0.414	-0.005(-0.059, 0.050)	0.858	-0.008(-0.071, 0.055)	0.757
	Q3	0.135(0.083,0.187)	<0.001	0.006(-0.045, 0.056)	0.82	0.009(-0.044, 0.062)	0.716	0.008(-0.055, 0.071)	0.751
	P for trend		<0.001		0.805		0.712		0.744
FT4	Q1	ref		ref		ref		ref	
	Q2	-0.208(-0.513, 0.096)	0.173	-0.152(-0.436, 0.132)	0.281	-0.154(-0.432, 0.124)	0.256	-0.144(-0.478, 0.190)	0.318
	Q3	-0.636(-0.902, -0.370)	<0.001	-0.542(-0.779, -0.305)	<0.001	-0.544(-0.786, -0.302)	<0.001	-0.492(-0.803, -0.181)	0.010
	P for trend		<0.001		<0.001		<0.001		0.007

adjusted 1: adjusted for age, race, sex.

adjusted 2: adjusted for age, race, sex, marital status, education level, family annual income, BMI.

adjusted 3: adjusted for age, race, sex, marital status, education level, family annual income, BMI, Hypertension, stroke, diabetes, smoke, alcohol using.

PA, Physical activity; TT4RI, Thyrotroph Thyroxine Resistance Index; TSHI, Thyroid Stimulating Hormone Index; TFQI, Thyroid Feedback Quantile-based Index; TSH, Thyroid Stimulating Hormone; FT3, Free triiodothyronine; FT4, Free Thyroxine.

### 3.3 Association between thyroid hormone indexes and dysthyreosis

Subsequently, we explored the relationship between the key thyroid hormone indexes including TFQI and TSHI, and the dysthyreosis occurrence via the logistics regression analysis. The results showed that the association between the TFQI and dysthyreosis occurrence was statistically significant both in crude model and 3 adjusted models (all P<0.05). However, there was no obvious relationship between the TSHI and the dysthyreosis occurrence (**Table 3**).

**Table 3:** The association between TFQI/TSHI and dysthyreosis in the whole population by logistic regression analysis (n=2118).

independent variable	dependent variable	crude model		adjusted model 1		adjusted model 2		adjusted model 3	
		95%CI	P	95%CI	P	95%CI	P	95%CI	P
TFQI	dysthyreosis	9.901(2.880,34.033)	<0.001	10.042(2.716,37.126)	0.001	10.232(2.509,41.739)	0.003	10.567(1.634,68.340)	0.025
TSHI	dysthyreosis	0.771(0.282,2.109)	0.602	0.759(0.290,1.987)	0.561	0.767(0.296,1.987)	0.559	0.792(0.245, 2.560)	0.610

adjusted 1: adjusted for age, race, sex.

adjusted 2: adjusted for age, race, sex, marital status, family annual income, education level, BMI.

adjusted 3: adjusted for age, race, sex, marital status, family annual income, education level, BMI, smoking, alcohol using, hypertension, diabetes, stroke.

TFQI, Thyroid Feedback Quantile-based Index; TSH, Thyroid Stimulating Hormone.

### 3.4 Association among the PA total MET, TFQI, and dysthyreosis

**Figure 2:**
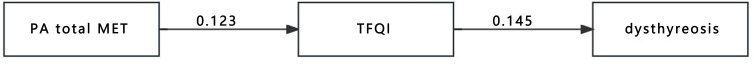
Path among the PA total MET, TFQI, and dysthyreosis.

Based on the above results, we speculated that PA total MET may affected thyroid function by TFQI. Hence, we performed the path analysis to verify the conjecture. The path analysis showed that the PA total MET effected dysthyreosis occurrence by TFQI (r (PA total MET ~ TFQI) =0.123, P<0.05; r (TFQI ~ dysthyreosis) =0.145, P<0.05, **Table 4**, **Figure 2**).

**Table 4:** Path analysis among the PA total MET, TFQI, and dysthyreosis.

X → Y	Standardized coefficient	SE	CR	P
PA total MET → TFQI	-0.123	0.000	-5.709	P<0.001
TFQI → dysthyreosis	0.145	0.019	6.747	P<0.001

PA, Physical activity; MET, Metabolic Equivalent Task; TFQI, Thyroid Feedback Quantile-based Index; SE, standard error; CR, critical ratio.

### 3.5 Association between the PA total MET and dysthyreosis

**Figure 3:**
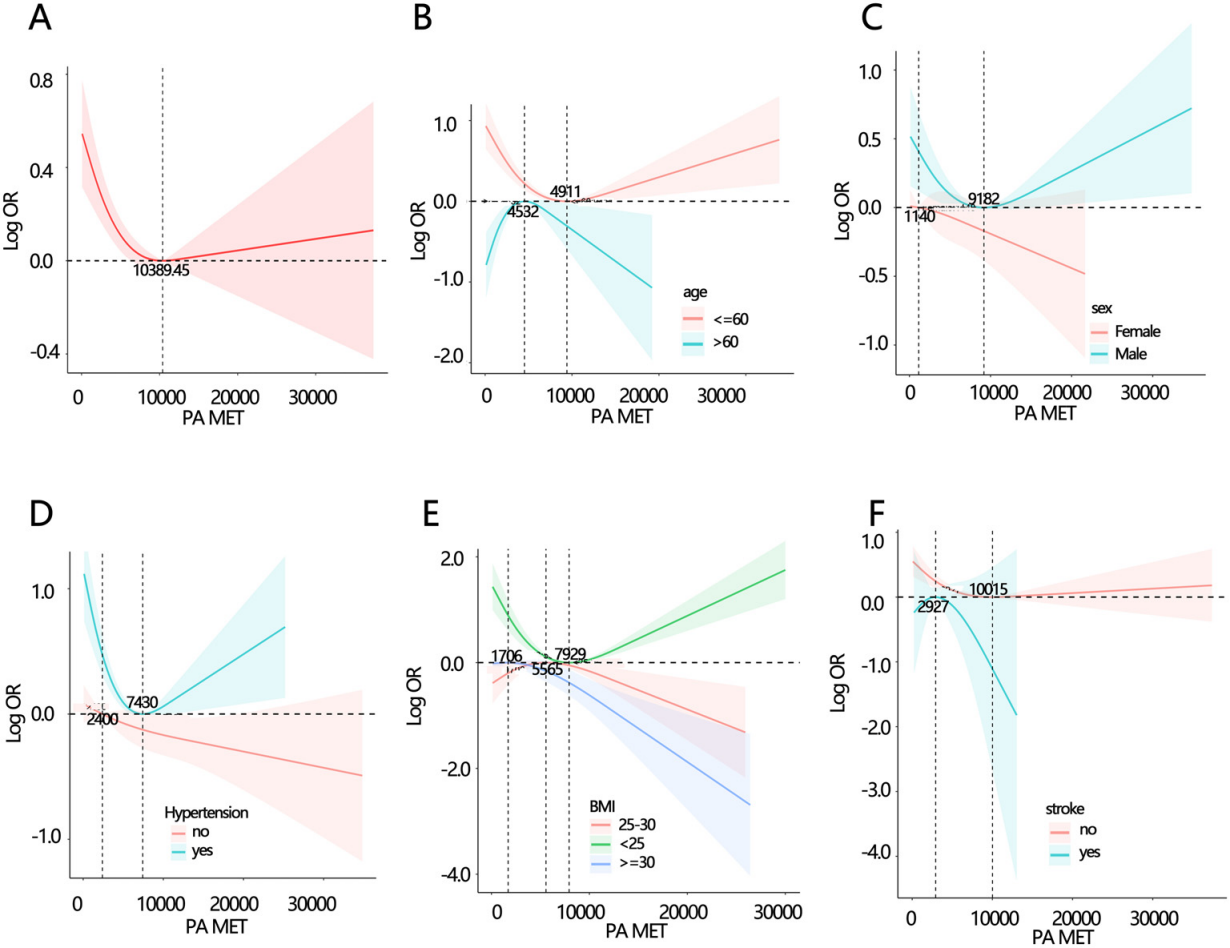
Spline analyses of PA total MET with dysthyreosis in participants and subgroup patients. **(A)** all participants; **(B)** age subgroup; **(C)** sex subgroup; **(D)** hypertension subgroup; **(E)** BMI subgroup **(F)** stroke subgroup.

We further explored the association between the PA total MET and dysthyreosis, the spline analyses indicated that the PA total MET was non-linearly associated with dysthyreosis (P for non-linearity<0.05, **Figure 3A**). Then, the stratified analysis (**Figures 3B-F**) grouped by age, sex, hypertension, BMI, and stroke further explored the relationship between PA total MET and dysthyreosis. The results showed that the non-linearity between the PA total MET and dysthyreosis was affected by age, hypertension, BMI, and stroke.

## 4 Discussion

This study revealed vital findings on the basis of the NHANES database. First, we discovered that PA affected the function of the thyroid via sensitivity to thyroid hormones. Second, there was a non-linear relationship between the PA and dysthyreosis, which was affected by age, hypertension, BMI, and stroke.

A previous study reported that regulated PA played a significant role in maintaining psychological and physical health (22). In this study, we found that PA was associated with the TFQI, which may influence the degree of negative feedback between the thyroid hormones and TSH in the central pituitary (12). Physically active people usually have beneficial cardiac metabolic characteristics and fat consumption (23). Fat metabolism can lead to decreased blood leptin levels. Leptin, which is produced and secreted in peripheral adipose tissue, provides feedback at the paraventricular nucleus of the hypothlamus to stimulate signal transducers and activators of transcription 3 phosphorylation, directly stimulating TRH expression (24, 25). Moreover, it also stimulates thyrotropin-releasing hormone (TRH) by an indirect pathway that it stimulates multifunctional premise protein proopiomelanocortin (POMC) and generates α-melanocyte-stimulating hormone (α-MSH) through inhibition of neuropeptide Y and spiny mouse-associated proteins. α-MSH stimulates cyclic-AMP response binding protein in TRH neurons (24). Further impact the thyroid hormone sensitivity by the hypothalamic–pituitary–thyroid axis (24). In addition, PA also affects the secretion of adrenaline, which interacts with thyroid hormones via adrenergic signaling (26, 27). In summary, PA affected the TFQI by influencing the production and secretion of other hormones including adrenaline, leptin, and dopamine that had a function of a point of central regulation in the hypothalamic–pituitary–thyroid axis (28).

The study by Oscar Hernando Roa Dueñas et al. indicated that there was no association between the endogenous thyroid hormone level and total PA, which was consistent with our study (29). No obvious association between the PA and TSH or FT4 was found as well. However, we found that PA may affect the thyroid function via TFQI by the path analysis. TFQI is an empirical joint distribution of FT4 and TSH, and they were the main physiological indicators for the public to evaluate thyroid function. The advantage is that TFQI does not produce an extreme value in the case of thyroid dysfunction and is more stable than FT4 and TSHI (12). Therefore, the TFQI was the important bridge between PA and thyroid function in this study.

Most studies have shown that thyroid hormone was associated with age and chronic metabolic diseases. For instance, higher FT4 levels in individuals increased the risk of atrial fibrillation (30), also was associated with cardiovascular disease and stroke in middle-aged and elderly people (31, 32). Therefore, we speculated that the relationship between PA and dysthyreosis may be affected by these factors. The subgroup analysis indicated that non-linear relationship between the PA and dysthyreosis was affected by age, hypertension, BMI, and stroke. The finding could provide a PA-related suggestion for those different populations.

Although our results indicated the association between PA and dysthyreosis, it had some disadvantages. First, the cross-sectional study could not assess the causal relationship between the variables. Hence, we made up for this defect through path analysis. Second, the PA was counted via a self-reported questionnaire, which may be biased due to the information of recollection. Third, Participants with thyroid function disorder were not further studied according to disorder subtypes.

## 5 Conclusion

In summary, we found that PA affected the function of thyroid via sensitivity to thyroid hormones. The results of the present study played an important role in the research of public health because these findings provided a reference for the mechanism research of exercise and thyroid function. At the same time, this study also provided a new insight into the relationship between MET and dysthyreosis.

## Data Availability

The raw data supporting the conclusions of this article will be made available by the authors, without undue reservation.
